# Up-regulation of fas reverses cisplatin resistance of human small cell lung cancer cells

**DOI:** 10.1186/1756-9966-29-49

**Published:** 2010-05-14

**Authors:** Wei Wu, Hai-dong Wang, Wei Guo, Kang Yang, Yun-ping Zhao, Yao-guang Jiang, Ping He

**Affiliations:** 1 Department of Thoracic Surgery, Institute of Surgery Research, Daping Hospital, Third Military Medical University, Chongqing 400042, PR China; 2 Department of Thoracic Surgery, Southwest Hospital, Third Military Medical University, Chongqing 400038, PR China

## Abstract

**Background/Aim:**

Fas/FasL system is a major regulator of apoptosis. The mechanisms by which Fas mediates cisplatin resistance remain unclear. The aim of this study is to explore the effect of Fas over-expression on cisplatin resistance of small cell lung cancer cells and its possible mechanisms.

**Materials and methods:**

Fas was over-expressed in H446/CDDP cells by infection with the adenoviruses containing Fas. Sensitivity of Fas-overexpressed H446/CDDP cells to cisplatin was evaluated using MTT assay. Expressions of Fas, GST-π and ERCC1 were detected by RT-PCR and Western blot analysis. Apoptosis rate was examined by FACS.

**Results:**

Over-expression of Fas in H446/CDDP cells significantly decreased the expressions of GST-π and ERCC1 at mRNA and protein levels, and increased the cell apoptosis. Furthermore, up-regulation of Fas significantly decreased the tolerance of H446/CDDP cells to cisplatin.

**Conclusion:**

Over-expression of Fas reverses drug resistance of H446/CDDP cells, possibly due to the increased cell sensitivity to apoptosis and the decreased expressions of GST-π and ERCC1.

## Background

Small cell lung cancers (SCLC) are well known for their initial sensitivity to chemotherapeutic agents and thereafter frequent recurrence when tumors exhibit drug resistance. Cisplatin, formally known as cis-diamminedichloroplatinum (II) (CDDP), is a metal-base oncolitic agent that binds to the nucleophilic sites of DNA resulting in changes in DNA synthesis and cell death [1]. For this reason, cisplatin is commonly recommended for chemotherapeutical treatment of SCLC. However, many patients with SCLC exhibit drug resistance, which hampers the outcomes of cisplatin treatment.

It is well known that a number of cellular adaptations, including reduced drug uptake, drug inactivation by glutathione and other antioxidants, increased levels of DNA repair enzymes or DNA tolerance, and altered drug targets could cause resistance to CDDP, which may contribute to the poor survival rate of SCLC patients. Dysfunction of apoptotic signal transduction pathway of malignant cells can also cause drug resistance. For example, down-regulation of pro-apoptotic genes such as Bax and Fas/FasL and up-regulation of anti-apoptotic genes such as Bcl-2 has been involved in drug resistance. Fas, a 45 kDa type I transmembrane protein, is expressed on cell membranes of varieties of normal cells and malignant cells including lung cancer cells [2,3]. Its ligand, FasL, is expressed on the cell membrane of activated T lymphocytes and some malignant cells [4,5]. After trimerization of Fas on the cell membrane by extracellular FasL [6], Fas-associated death domain (FADD) and caspase 8 bind to the intracellular death domains of Fas and induce a death signal in the cells [7], leading to the activation of a cascade of caspases and eventually to cell death. Since FasL can induce apoptosis in Fas-expressing malignant cells, the Fas/FasL system plays an important role in T cell-mediated cytotoxic reaction and malignant cell-mediated autocrine suicide or paracrine death against malignant cells. On the other hand, malignant cells can avoid being killed by down-regulating Fas expression. It has been demonstrated that cisplatin-resistant lung cancer cells express low level of Fas, and correspondingly, their apoptosis decreases significantly. Some reports have correlated multidrug resistance (MDR) with the decreased Fas expression and resistance to Fas-mediated apoptosis. Fas-resistant cells were resistant to chemotherapeutic drug treatment, which is presumably due to the disruption of pathways responsible for the induction of cell death by chemotherapeutic drugs [8].

Many agents can induce the expression of Fas, and thus promote the apoptosis of malignant cells. Cisplatin can enhance some solid tumors or leukaemic cell surface expression of Fas [9-11] via the activation of the acid sphingomyelinase (aSMase) and the generation of ceramide at the plasma membrane. Up-regulating the expression of melanoma differentiation-associated gene-7/interleukin-24 (MDA-7/IL-24) can enhance the expression of Fas activated by cisplatin. Cisplatin can also enhance MDA-7/IL-24 toxicity via activation of the extrinsic pathway and de novo ceramide synthesis [12]. Bruno Segui et al proposed that it might be a way to treat cancer by enhancing the expression of Fas and promoting the apoptosis of tumor cell [13]. But in cisplatin-resistant human squamous cell carcinomas of the head and neck (SCCHN) cells, although the expression of Fas was enhanced by cisplatin or IFN-γ, the cisplatin sensitivity cannot be restored by agonistic Fas-antibodies [14]. However, the agonistic Fas-antibodies could restore the cisplatin sensitivity in other entities like bladder carcinomas [15]. Many factors may be involved, including that: 1. High expression of drug-resistance genes such as glutathione S-transferase π (GST-π) and excision repair cross-complementing-1 (ERCC1) may be the major mechanism of drug resistance, and Fas-FasL system may be a minor one; 2. In SCCHN, the expression of Fas activated by cisplatin is p53-independent and may be ineffective activation, which was in contrast to many other solid tumors, where the antiproliferative effect of anticancer drugs is mediated at least in part by the Fas-FasL system via p53-dependent mechanisms [16].

It is still obscure whether up-regulation of Fas expression can reverse cisplatin resistance, increase cisplatin-induced apoptosis, and alter the expression of any drug-resistant gene in human SCLC cells. To explore the possible role of Fas on cisplatin resistance in SCLC cells, we established a cisplatin-resistant SCLC cell line (H446/CDDP), and constructed adenovirus vector containing Fas gene. By overexpressing Fas, we investigated the role of Fas in cisplatin sensitivity and apoptotic rate of SCLC cells. We also examined the levels of GST-π and ERCC1, given their involvement in drug binding/inactivation and nucleotide excision repair (NER). Our results indicate that up-regulation of Fas could reverse cisplatin resistance of human SCLC cells by decreasing the expressions of GST-π and ERCC1 and increasing Fas-mediated apoptosis.

## Methods

### Cell lines and culture conditions

Cisplatin was obtained from Ebewe Arzneimittel Ges.m.b.H. (Austria). Human SCLC cell line H446 was obtained from Academy of Military Medical Science (Beijing, China) and maintained in RPMI 1640 (Trace, Melbourne, Australia) supplemented with 10% fetal bovine serum (FBS), 100 U/ml penicillin, and 100 μg/ml streptomycin at 37°C, in a humid atmosphere of 5% CO2/95% air. Exposing them to gradually increasing concentrations of cisplatin (up to 30.8 μg/ml) induced in vitro cisplatin-resistant cells. The obtained cell sublines H446/CDDP were maintained in the absence of drug, and its drug resistance was stabilized by 30.8 μg/ml CDDP treatment for 4 days every 6 weeks. H446/CDDP is 39.0 times as resistant to cisplatin as its parental cell line. Cells from exponentially growing cultures were used for all experiments.

### Adenovirus vector construction and gene transduction

Total RNA was extracted from H446 cells and first strand of cDNA was synthesized, the open reading frame (ORF) of human Fas gene was cloned using the primers with restriction endonuclease site as following: up primer 5' GGGGTACC ATGCTGGGCATCTGGACCCTC 3'(*Kpn *I) and 5' GCTCTAGA TCACTCTAGACCAAGCTTTGG 3' (*Xba *I). PCR reaction was performed with 5 min of initial denaturation at 94°C, 30 cycles of 30 s denaturation at 94°C, 30 s annealing at 61°C, 45 s extension at 72°C, and finally 10 min extension at 72°C. The PCR product of target fragment about 1013 bp was purified and ligased into T-vector, and the positive bacteria colonies were screened for sequencing by ampicillin resistance and blue-white screening with X-gal and IPTG. The Fas gene was subcloned to pAdTrack-CMV plasmid (a gift from Gang Huang, Third Military Medical University, Chongqing, China) and recombinants of pAdTrack-CMV-Fas were generated by transformation the shuttle plasmid linearized with *Pme *I to BJ5183 cells with the adenoviruses backbone plasmid for homologous recombination. The recombinant adenoviruses were packaged and propagated in 293 cells. Viral titers were determined by standard plaque assay after the Fas adenoviruses concentrated by CsCl ultracentrifugation using a standard method [17].

H446/CDDP cells were transfected with 50 multiplicity of infection (MOI) of adenoviruses in serum free RPMI and maintained in complete medium at 37°C until post-transfection day 3. The transfectants overexpressing Fas were obtained and designated as H446/CDDP/Fas. H446/CDDP cells transfected with empty adenoviruses were indicated as H446/CDDP/Empty and used as negative control in all assays.

### Conventional RT-PCR analysis

On post-transfection day 3, total RNAs were isolated from H446/CDDP, H446/CDDP/empty, and H446/CDDP/Fas cells using TRIzol reagent (TianGen, Beijing, China) and subsequently used for semiquantitative PCR. RT was performed with 1 μg of total RNA from each sample using oligo(dT) 18 primers and 200 units of SuperScript II RT (Life Technologies Inc., Gaithersburg, Md., USA) for cDNA synthesis. cDNA amplification was conducted in 20 μl solution containing 2 μl of diluted cDNA, 10 pmol primer pairs for Fas, GST-π, ERCC1 and GAPDH, respectively, and 10 μl of Taq PCR Master mix (TianGen, Beijing, China). The PCR consisted of initial denaturation at 94°C for 5 min, followed by 30 reaction cycles (30 seconds at 94°C, 30 seconds at 61°C, and 30 seconds at 72°C) and a final cycle at 72°C for 10 min. Primers used in PCR were listed in Table 1. GAPDH was used as internal control. All PCR products were electrophoretically separated on ethidium bromide-stained agarose gel and visualized with UV light.

**Table 1:** PCR primer sequences and product sizes.

**Primers**^**a**^	Oligonucleotide Sequences	Product Size (bp)	PCR Cycles
Fas	F: 5'GTCCAAAAGTGTTAATGCCCAAGT 3'	232	30
	R: 5'ATGGGCTTTGTCTGTGTACTCCT 3'		
GST-π	F: 5' CCGCCCTACACCGTGGTCTAT 3'	260	30
	R: 5' GCTGCCTCCTGCTGGTCCTT 3'		
ERCC1-2	F: 5' ACGCCGAATATGCCATCTCAC 3'	292	30
	R: 5' AGCCGCCCATGGATGTAGTCT 3'		
GAPDH	F: 5' ACCCATCACCATCTTCCAGGAG 3'	159	30
	R: 5' GAAGGGGCGGAGATGATGAC 3'		

^a ^All primers were designed using genetool software.

### Real-time quantitative PCR (RT-qPCR)

RT-qPCR was performed with ABI 7500 Thermal Cycler and SYBR Green qPCR kit (Toyobo, Japan). PCR reactions were prepared in low-profile microplates with each well containing 10 μl of master mix, 2 μl of diluted cDNA, 10 pmol each of primers listed in Table 1 for Fas, GST-π, ERCC1 and control GAPDH, respectively, in a 20 μl reaction volume. The PCR consisted of initial denaturation at 94°C for 5 min, followed by 30 reaction cycles (30 seconds at 94°C, 30 seconds at 61°C, and 30 seconds at 72°C) and a final cycle at 72°C for 10 min. Amplifications were performed in triplicate according to the cycling protocol provided by the manufacturer. Gene expression was expressed as 2^-ΔΔ(Ct) ^[18], where Ct is cycle threshold, Δ(Ct) = Ct of tested gene - Ct of GAPDH; ΔΔ(Ct) = Δ(Ct) of sample 1-Δ(Ct) of sample 2.

### Western blot analysis

The mouse anti-human Fas (cat. sc-74540), GST-π (cat. sc-58368) and rabbit anti-human ERCC1(cat. sc-10785) antibodies and horseradish peroxidase(HRP)-conjugated goat anti-rabbit and goat anti-mouse immunoglobulin G (IgG) were obtained from Santa Cruz Biotechnology (Santa Cruz, Calif., USA). 5 × 10^6 ^H446/CDDP Cells were seeded into 100 mm plates, incubated for 24 h at 37°C, and then transfected with 50 MOI of adenoviruses. On post-transfection day 3, H446/CDDP, H446/CDDP/Fas, and H446/CDDP/empty cells were washed three times with cold phosphate buffered saline (PBS) and then lysed in RIPC buffer (0.5 M NaCl, 0.5% NP-40, 20 mM Tris-HCl pH 8, 1 mM PMSF). The protein levels were determined using an ECL kit ((Amersham Pharmacia, Uppsala, Sweden). Total cellular proteins were diluted 2-fold into SDS-PAGE loading buffer (NEB). The samples were heated to 95°C for 5 min before an aliquot of 20 μl of each diluted assay sample, containing approximately 50 ug of total protein, was loaded onto a 6-12% Tris-glycine polyacrylamide gel (Invitrogen). Proteins were resolved by SDS-PAGE and then transferred to a 0.45 μm nitrocellulose membrane (Whatman). The membrane was blocked with 5% nonfat dry milk in Tris-buffered saline (50 mM Tris-HCl, pH 7.5, 150 mM NaCl) supplemented with 0.2% Tween 20 and 0.05% Triton X-100 (TBSTT). The membrane was probed with the primary antibody at 1:700 dilution in TBSTT supplemented with 2% nonfat dry milk. After an overnight incubation at 4°C, the membrane was washed and incubated at room temperature for 2 h with a goat anti-rabbit or mouse HRP-linked IgG antibody (1:700 dilution in TBSTT with 2% dry milk). Binding of the antibody was detected by chemiluminescence with the Phototope-HRP Western Blot Detection System (CST).

### *In vitro *drug sensitivity assay

Drug sensitivity was evaluated using 3-(4,5-dimethylthiazol-2-yl)-2,5-diphenyl-tetrazolium bromide (MTT) assay. Briefly, on post-transfection day 3, the transfected cells and control cells were seeded into 96-well plates with 10^3 ^cells per well and incubated overnight. Cells were then incubated with CDDP in different concentrations (5, 10, 15, 20, 25, 30, 35, 40, 45, and 50 μg/ml). After 72 h of incubation, 20 μl of 5 mg/ml MTT (Sigma Chemical Co., St Louis, MO) in PBS was added to each well, followed by incubation for 4 h at 37°C. The formazan crystals were dissolved in 50 μl of dimethyl sulfoxide (DMSO). The optimal density was determined with microculture plate reader (Becton Dickinson Labware, Lincoln Park, NJ) at 570 nm. Absorbance values were normalized to the values obtained for control cell to determine the proportion of survival [19]. The IC50 values were the drug concentrations causing a 50% reduction in the optical density. The experiments were performed in triplicate, and expressed as the mean values of three experiments.

The relative resistance was calculated by the following formula:

### Apoptosis analysis

On post-transfection day 3, cells were resuspended in 100 μl binding buffer at a concentration of 1 × 10^6^/ml after washing twice with cold PBS and mixed with 5 μl Annexin V-FITC (PharMingen) and 10 μl of 20 μg/ml propidium iodide (Sigma) at room temperature for 15 min. Samples were diluted with 400 μl binding buffer and analyzed by fluorescence activated cell sorting (FACS) using the protocol provided by the manufacturer (ClonTech, Palo Alto, Calif., USA). The apoptotic rate was calculated as the mean fluorescence intensity.

### Statistical analysis

The data are expressed as the mean ± SEM. Each experiment was repeated at least three times. Bands from Western blots were quantified by Quantity One software (Bio-Rad). The differences among means were examined with ANOVA followed by post-hoc test using SPSS 11.0 software (Chicago, Ill., USA). A p value less than 0.05 was considered as statistical significance.

## Results

### RT-PCR and Western blots

**Figure 1:**
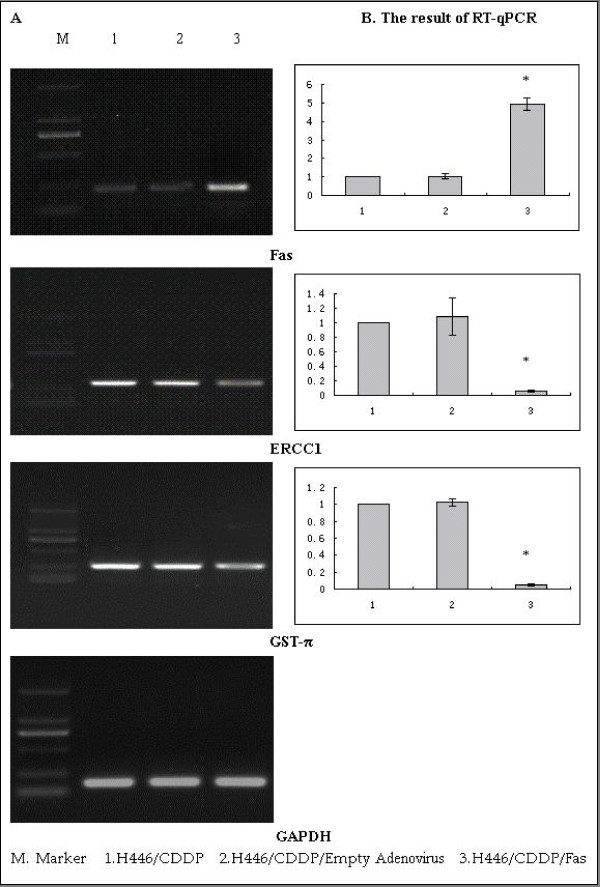
**The expression of Fas, ERCC1, GST-π and GAPDH detected by RT-qPCR**. GAPDH was used as an internal control. Upregulation of Fas led to a significant decrease in ERCC1 and GST-π. * p < 0.01 *vs *H446/CDDP/Empty and H446/CDDP cells.

**Figure 2:**
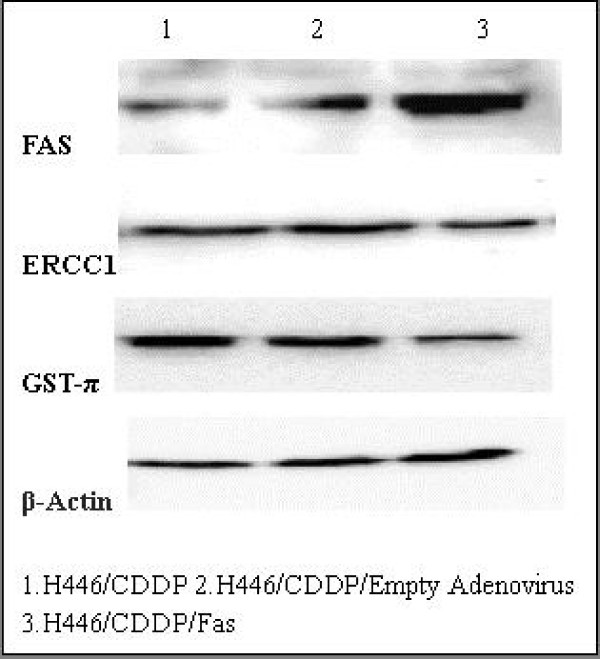
**The expression of Fas, ERCC1, and GST-π detected by Western blots**. β-actin was used as an internal control. Upregulation of Fas caused the downregulation of ERCC1, and GST-π.

Both mRNA and protein levels of Fas were significantly lower in H446/CDDP and H446/CDDP/Empty cells compared with those in H446/CDDP/Fas cells (p < 0.01), indicating that Fas was successfully transduced into and expressed in H446/CDDP cells. Over-expression of Fas effectively down-regulated ERCC1 and GST-π in both mRNA and protein levels (p < 0.01) compared with the control cells (Figs. 1 and 2).

### Effect of Fas on cisplatin resistance

To explore the roles of Fas in cisplatin resistance of SCLC, MTT assays were performed. 72 h after exposure to CDDP, the 50% inhibitory concentration (IC_50_) of CDDP in H446/CDDP/Fas was 7.6 ± 0.46 μg/ml, significantly lower than 30.8 ± 0.92 μg/ml and 29.7 ± 0.26 μg/ml in H446/CDDP and H446/CDDP/Empty, respectively (p < 0.01). In other words, H446/CDDP/Fas cells showed a 3.9-fold decrease in resistance to CDDP compared with H446/CDDP/Empty cells, suggesting that up-regulation of Fas could inhibit the cisplatin-resistant phenotype of SCLC.

### Effect of Fas on cell apoptosis

**Figure 3:**
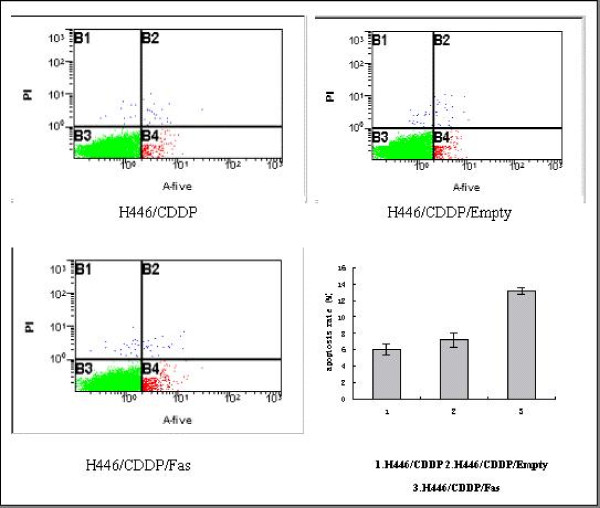
**The apoptotic rate of H446/CDDP, H446/CDDP/Empty, and H446/CDDP/Fas cells**. Compared to H446/CDDP and H446/CDDP/Empty cells, H446/CDDP/Fas cells had a significantly increased apoptotic rate (p < 0.01).

The apoptosis rates in H446/CDDP, H446/CDDP/Empty and H446/CDDP/Fas cells were 6.02 ± 0.70%, 7.19 ± 0.89% and 13.17 ± 0.40%, respectively. Compared to H446/CDDP and H446/CDDP/Empty cells, H446/CDDP/Fas cells showed a significantly lower apoptotic rate (p < 0.01, Fig. 3), suggesting that the up-regulation of Fas promoted the apoptosis in H446/CDDP cells.

## Discussions

As one of the most widely used platinum-containing anticancer drugs, CDDP is believed to induce tumor cell death as a result of the formation of CDDP-DNA adducts, which inhibits DNA replication and transcription [20]. The presence of intrinsic or acquired resistance to CDDP in cancer cells limits curative effects of chemotherapy. Therefore, understanding the precise mechanisms of CDDP resistance and reversing it would provide new strategies for cancer therapy.

The balance of Fas/FasL interaction between the host immune system and malignant cells may be crucially involved in determining sensitivity or resistance towards chemotherapy. In several malignant cell lines, including SCLC cell lines, commonly used chemotherapeutic drugs have been shown to induce Fas expression [21]. Cisplatin can promote apoptosis of malignant cells by inducing Fas expression, which is one of the mechanisms of cisplatin killing the malignant cells. For instance, cisplatin could up-regulate expressions of Fas and FasL, activate caspase 8 pathways and induce apoptosis in uterine cervix cancer cells [22]. Matsuzaki I et al [23] found that cisplatin could induce Fas expression in esophageal cancer cell lines and enhance cytotoxicity in combination with LAK cells. Lan F. Qin et al [24] found that cisplatin could induce expression of Fas in hepatoma cells, which was correlated with the appearance of cisplatin-induced apoptosis. But the cisplatin-resistant malignant cells usually express low level of Fas, and correspondingly, the apoptosis of malignant cells decreases significantly. Fas-resistant cells are resistant to chemotherapeutic drug treatment, which is presumably due to the disruption of the pathway responsible for cell death induced by chemotherapeutic drugs [25]. In our study, the enhanced mRNA and protein expressions of Fas in cisplatin-resistant SCLC cells correspondingly increases SCLC cell apoptosis.

The mechanisms of resistance to CDDP are multifactorial, and many genes or gene products have been reportedly responsible for CDDP resistance [26]. Cisplatin is most efficiently removed from transcribed areas within DNA, and gene-specific repair efficiency of cross-links correlates with cisplatin resistance [27]. Platinum damage is repaired primarily by the nucleotide excision repair (NER) system (particularly ERCC1 and ERCC1/XPF) and the related genes XPA and BRCA1 [28,29]. Previous studies have found that increased expression of ERCC1, an important NER protein, is correlated with CDDP resistance. For instance, expression of ERCC1 has been shown to increase the resistance to platinum treatment in patients with ovarian cancer [30]. Similarly, non-small cell lung carcinoma (NSCLC) displayed a correlation between CDDP resistance and ERCC1 levels [31-33]. Testicular cancer, generally very responsive to CDDP, has low level of ERCC1, providing further correlative evidence for the importance of ERCC1 in CDDP resistance [34]. Given its involvement in the NER DNA repair pathway, we paid special attention to ERCC1. A previous study has proved that the suppression of ERCC1 expression in human cancer cells leads to an increased sensitivity to CDDP, and ERCC1 has been presumed to be an attractive target to confer increased cellular sensitivity to CDDP-based chemotherapy [35]. The results of this study suggest that the expression of ERCC1 is significantly down-regulated with the transfection of Fas in H446/CDDP cells, which may contribute to the decreased resistance to CDDP.

Increased glutathione (GSH) may cause resistance by binding/inactivating cisplatin, enhancing DNA repair, or reducing cisplatin-induced oxidative stress [36]. Glutathione-*S*-transferase (GST), particularly GST-π [37,38], may augment drug resistance by catalyzing GSH-drug binding. Clinically, GST-π gene amplification [39], immunostaining [40], and plasma levels [41] have been correlated with cisplatin resistance, suggesting that platinum detoxification by GSH and GST may be clinically important. The results of this study suggest that the expression of GST-π is significantly down-regulated with the transfection of Fas in H446/CDDP cells, which may contribute to the decreased resistance to CDDP.

## Conclusion

Our results show that Fas gene transduction can reverse the multidrug resistance (MDR) of human drug resistant SCLC cell H446/CDDP, for which the enhanced cell sensitivity to apoptosis and decreased expression of GST-π and ERCC1 may be responsible. Although the biological function of Fas in SCLC needs to be further investigated, the present results of our study provide a framework for the illumination of the resistance to CDDP mediated by Fas, and will aid in the effective use of CDDP in SCLC treatment.

## Competing interests

The authors declare that they have no competing interests.

## Authors' contributions

WW: Participated in research design, the writing of the paper, the performance of the research and data analysis. HDW: Participated in research design, the performance of the research and data analysis. WG: Participated in research design. KY: Participated in research design, the performance of the research and data analysis. YPZ: Participated in research design. YGJ: Participated in research design, the writing of the paper, the performance of the research and data analysis. PH: Participated in the writing of the paper and data analysis. There is no conflict of interest for each author. All authors read and approved the final manuscript.
